# Entosis enables a population response to starvation

**DOI:** 10.18632/oncotarget.20066

**Published:** 2017-08-09

**Authors:** Jens C. Hamann, Michael Overholtzer

**Affiliations:** Cell Biology Program, Memorial Sloan Kettering Cancer Center, New York, NY, USA

**Keywords:** entosis, cell-in-cell, cannibalism, cell competition, starvation

Cell death isn’t just about apoptosis anymore. In the last decade, numerous alternative mechanisms have emerged, including regulated forms of necrosis, as well as entosis, a unique mechanism that is executed as a cell murder rather than cell suicide [1]. Hamann et al. now identify glucose starvation as a key inducer of this non-cell-autonomous form of cell death [2].

Cells that undergo entosis are killed as a result of ingestion into their neighbors. They are not phagocytosed, but rather form junctions with their neighbors and then invade into them, ultimately becoming killed through a mechanism involving autophagy proteins and lysosomal enzymes [1, 3]. This is a competitive process that results in elimination of stiffer cells (“losers”) by softer ones (“winners”), an effect linked to control over entotic cell invasion by key controllers of cell tension such as Rho-GTPase, Rho-kinase, and myosin [4].

During entosis, the nutrients derived from ingested loser cells are scavenged by winners and used in metabolism [5]. This prompted our studies to investigate whether entosis induction, like autophagy, might be regulated by nutrient signaling pathways. By examining cell starvation responses, we indeed discovered that glucose signaling is a key regulator of entosis, and that starvation for glucose, as well as total starvation for amino acids, glucose, and serum, induces high levels of this process, linking for the first time nutrient signaling to this competitive mechanism of nutrient scavenging.

How does nutrient starvation induce entosisŒ Biophysical studies indicated that glucose withdrawal leads to a bimodal mechanical response in starved cell populations, where some cells become stiffer, and others softer, than normal, resulting in a mechanical differential between neighboring cells. Activity of the energy sensor AMP-activated protein kinase (AMPK), a known activator of autophagy, is required for the appearance of stiffer cells, and also for entosis, specifically in loser cells. Long-term glucose starvation selects for winners with lowered levels of AMPK activity, and these cells are capable of ingesting naïve cells at high rates in mixed populations, demonstrating selection for the ability to scavenge nutrients through this competitive mechanism.

**Figure 1:**
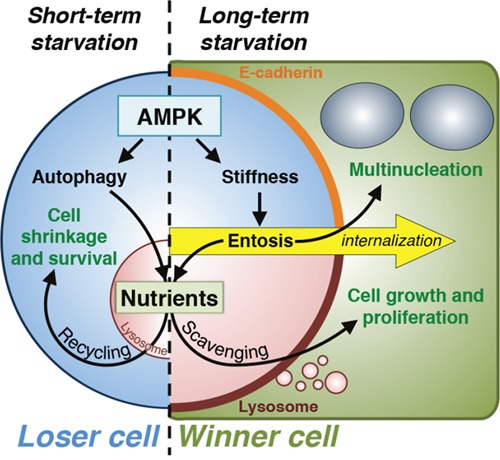
Nutrient scavenging through entosis Entosis can occur as a result of long-term starvation. Under short-term starvation stress (left side of figure), AMPK activates autophagy, resulting in intracellular nutrient recycling from the lysosome (red half-circle) that allows cells to survive. In contrast, during long-term starvation stress (right side), AMPK controls an increase in cell tension that, in cells maintaining adherens junctions through E-cadherin (orange), leads to entotic cell internalization (yellow arrow). Uptake of live loser cells (light blue) can result in cytokinesis failure during subsequent mitoses of winner cells (green), which generates multinucleated and aneuploid cell lineages (top right). Upon killing of internalized loser cells by winner cells, the resulting corpses are degraded within winner cell lysosomes, which supports the growth and proliferation of winners that scavenge nutrients (bottom right).

These findings link a key nutrient signaling pathway and known inducer of autophagy to regulation of entosis. While autophagy promotes the recycling of intracellular nutrients to support cell survival, entosis allows winner cells in a population to scavenge extracellular nutrients and accumulate the biomass necessary to support proliferation (Figure 1). Macropinocytosis has been shown to act similarly to allow cancer cells to scavenge extracellular proteins [5]. Entosis instead supplies bulk nutrients in the form of whole cells, where, on average, winner cells ingest two losers, providing a large nutrient supply that is well suited to support the outgrowth of selected cells in the population. Indeed, winners with ingested losers have a nearly 10-fold proliferative advantage, and entosis is required for population re-growth during long-term starvation.

Our findings identify entosis as a population-scale starvation response with parallels to cell competition occurring in developing tissues [6]. As mechanisms of cell competition select for the relative fitness of individual cells to promote tissue-scale fitness, entosis may similarly allow cell populations under extreme starvation stress to redistribute available nutrients to the fittest cells. Protection of the population may require sacrificing less-fit individuals, rather than rescuing those that may be under the most stress. In this model, regulation of loser cell mechanics by high levels of AMPK activity may be one signal that indicates a relative lack of fitness that could be sensed through cell junctions.

While we show that entosis is required for population re-growth, how this process links to the long-term proliferative capacity of winners is unknown, and may involve additional adaptations such as gluconeogenesis or other altered metabolic states that await discovery. Also, how AMPK signaling and autophagy pathway proteins coordinately regulate cell-autonomous (autophagy) versus non-cell-autonomous (entosis) processes will be interesting to explore in future studies. Autophagy proteins in this context control the digestion of extracellular nutrients through a pathway resembling LC3-Associated Phagocytosis, or LAP, first identified in macrophages [7]. We find that the autophagy protein-regulated death of ingested entotic cells is also controlled by glucose signaling, but in an AMPK-independent manner. How starvation impacts the death and degradation of ingested cells remains to be identified.

We have found that entosis is one of numerous non-apoptotic cell death mechanisms that has a unique property to promote competition between cells in a population. Recently, a regulated form of necrosis called ferroptosis was shown to have a different non-cell-autonomous effect, to spread from cell-to-cell [8]. How cell population dynamics in diseased or stressed tissues are controlled may reflect in part the percentages of different types of cell death that occur and their distinct cell properties. We find that glucose starvation induces apoptosis, necrosis, and entosis. Quantifying the percentages of mixed forms of cell death may be important for deciphering how different treatments influence cell selection. Entosis was first discovered in cell populations deprived of matrix adhesion [1], and another recent report has implicated the process of mitosis in controlling entosis, particularly for cells with disrupted polarity signals [9]. Further investigations to define the stressors that can induce this competitive process seem warranted.
